# *Withania somnifera*: Advances and Implementation of Molecular and Tissue Culture Techniques to Enhance Its Application

**DOI:** 10.3389/fpls.2017.01390

**Published:** 2017-08-09

**Authors:** Vibha Pandey, Waquar Akhter Ansari, Pratibha Misra, Neelam Atri

**Affiliations:** ^1^Department of Plant Molecular Biology, University of Delhi New Delhi, India; ^2^Department of Botany, Mahila Maha Vidhyalaya (MMV), Banaras Hindu University Varanasi, India; ^3^National Botanical Research Institute, Council of Scientific and Industrial Research Lucknow, India

**Keywords:** *Withania somnifera*, Ashwagandha, metabolites, withanolides, tissue culture, differentiation, transformation

## Abstract

*Withania somnifera*, commonly known as Ashwagandha an important medicinal plant largely used in Ayurvedic and indigenous medicine for over 3,000 years. Being a medicinal plant, dried powder, crude extract as well as purified metabolies of the plant has shown promising therapeutic properties. Withanolides are the principal metabolites, responsible for the medicinal properties of the plant. Availability and amount of particular withanolides differ with tissue type and chemotype and its importance leads to identification characterization of several genes/ enzymes related to withanolide biosynthetic pathway. The modulation in withanolides can be achieved by controlling the environmental conditions like, different tissue culture techniques, altered media compositions, use of elicitors, etc. Among all the *in vitro* techniques, hairy root culture proved its importance at industrial scale, which also gets benefits due to more accumulation (amount and number) of withanolides in roots tissues of *W. somnifera*. Use of media compostion and elicitors further enhances the amount of withanolides in hairy roots. Another important modern day technique used for accumulation of desired secondary metabolites is modulating the gene expression by altering environmental conditions (use of different media composition, elicitors, etc.) or through genetic enginnering. Knowing the significance of the gene and the key enzymatic step of the pathway, modulation in withanolide contents can be achieved upto required amount in therapeutic industry. To accomplish maximum productivity through genetic enginnering different means of *Withania* transformation methods have been developed to obtain maximum transformation efficiency. These standardized transformation procedues have been used to overexpress/silence desired gene in *W. somnifera* to understand the outcome and succeed with enhanced metabolic production for the ultimate benefit of human race.

## Introduction

**Figure 1:**
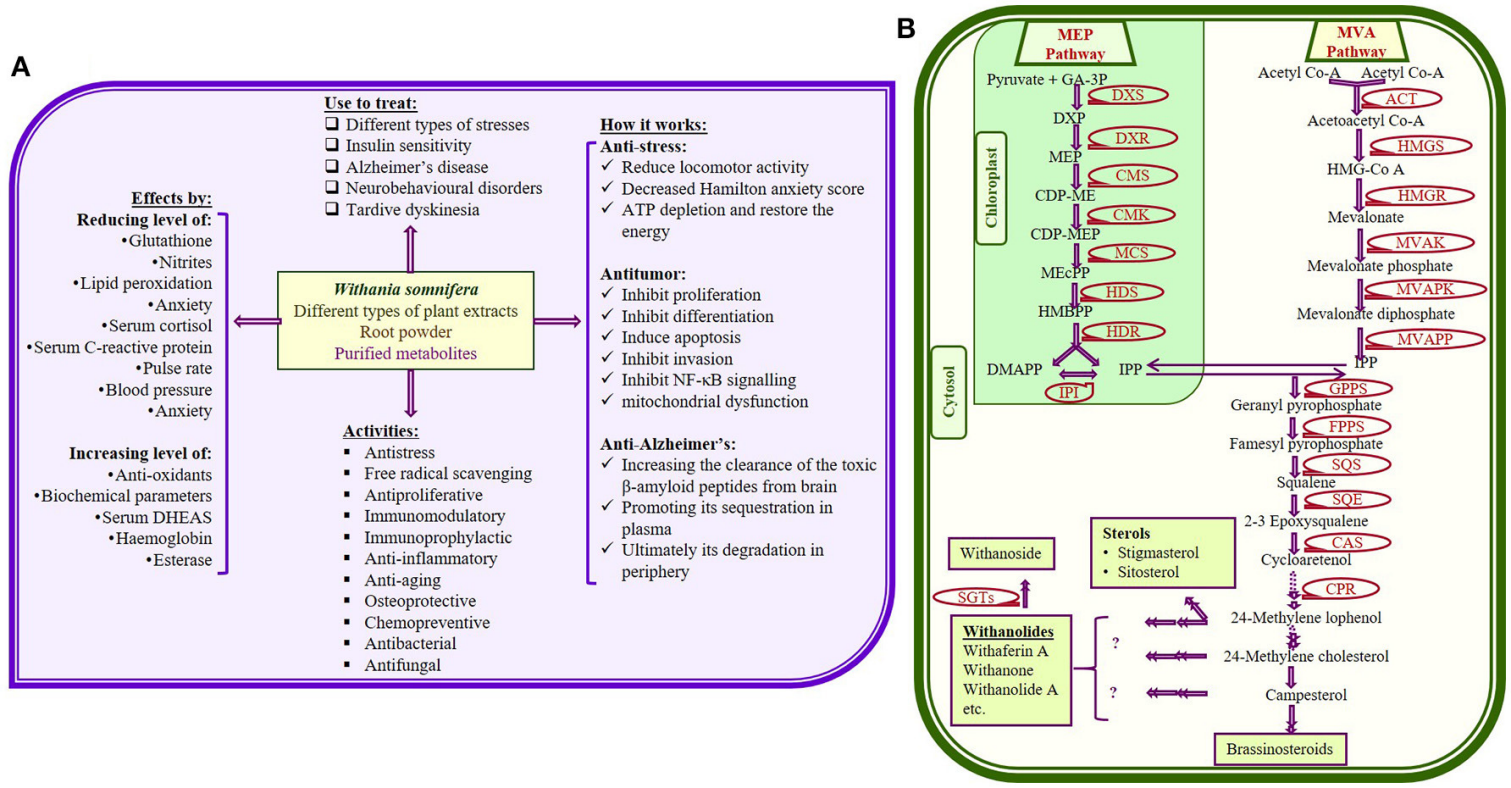
**(A)** Some important therapeutic uses of *Withania somnifera* with few proposed mode of actions (Dar et al., 2015); **(B)** Possible metabolic pathway for withanolides and glycowithanolides (withanosides) production (Senthil et al., 2010; Dhar et al., 2013; Sabir et al., 2013; Singh et al., 2015b) [GA-3P, glyceraldehyde-3-phosphate; MEP, 2-methyl- D-erythritol 4-phosphate; DMAPP, dimethylalyl pyrophosphate; IPP, isopentenyl pyrophosphate; IPPI, isopentenyl diphosphate isomerise; HMG-CoA, 3-hydroxy-3-methylglutaryl-coenzyme A; DXP, 1-deoxy-D-xylulose 5-phosphate; MVAPK, mevalonate phosphate kinase; MVAPP, diphosphomevalonate decarboxylase; CDP-ME, 4-diphospho-cytidyl-2-methyl-D-erythritol; CMS, 4-(cytidine-5-diphospho)-2-C-tmethyl-Derythritol synthase; CMK, 4-(cytidine-5-diphospho)-2-C-methyl-D-erythritol kinase, CDP-MEP, 2-C-methyl- D-erythritol-2-phosphate; MCS, 2-C-methyl-D-erythritol-2,4-cyclodiphosphate synthase; HDS, Hydroxy methyl butenyl 4- diphosphate synthase; HMBPP, Hydroxy methyl butenyl 4-diphosphate; HDR, Hydroxy methyl butenyl 4-diphosphate reductase].

*Withania somnifera* (Ashwagandha; Solanaceae family) is one of the most recognized and studied medicinal plants due to its wide distribution all around the world. *W. somnifera* has been used for over 3,000 years in indigenous medicine (Ayurvedic) system (Scartezzini and Speroni, 2000; Kumar and Kalonia, 2007; Tuli and Sangwan, 2009; Singh et al., 2015b). Several studies collectively provide metabolic insight of more than 200 primary and secondary metabolic components of *W. somnifera*. Significance of *Withania* in therapeutic world has been recognized due to maximum accumulation and diversified form of withanolide. All the identified variants of withanolides became interesting for researchers due to their beneficial effects for human body (Figure 1A; Kumar et al., 2007; Kulkarni and Dhir, 2008; Sharada et al., 2008; Mirjalili et al., 2009; Singh et al., 2010; Dar et al., 2015).

Biosynthesis of metabolites could be improved effectively through genetic engineering, which requires full information of all the genes/enzymes involve in biosynthetic pathway. Using the limited reports available on genes as well as enzymes of *W. somnifera*, researchers have prosposed possible metabolic pathway for the synthesis of different withanolides (Figure 1B; Senthil et al., 2010; Dhar et al., 2013; Sabir et al., 2013). Genes, enzymes as well as metabolites of respective metabolic pathway show differential pattern of expression according to the plant part, age, season, and other environmental factors. Optimization of various tissue culture techniques become very important to explore *W. somnifera* at different aspects, as plants obtained from fileds are not enough for all *in vitro* studies. Therefore, efficient tissue culture techniques like, micropropogation, regeneration, organogenesis, hairy root production, etc. have been established. Also, development of transgenic plants has been considered as the most economical way to improve the yield of therapeutic metabolites on large scale.

Present review recognizes the importance of *W. somnifera* and disscuss in detail genes/enzymes involved in the biosynthesis of secondary metabolites. The review also includes the significance of *in vitro* techniques in order to modulate the productivity of *W. somnifera* according to the desired final product. Suitable combinations of these findings create a very cooperative setting to modulate expression profile of various genes using different circumstances, results in synthesis of various secondary metabolites of *W. somnifera*.

## Proposed pathways for biosynthesis of withanolides: medicinal component of *W. somnifera*

Withanolide biosynthesis involves the key upstream metabolic step of isoprenogenesis using isoprenoid as precursor. Isoprenogenesis is known to proceeds through two different independent pathways; mevalonic acid (MVA) and methylerythritol phosphate (MEP; also called deoxyxylulose pathway, DOXP) pathway (Chaurasiya et al., 2007; Sangwan et al., 2007). These pathways occur in cytosol and plastid, respectively and ultimately synthesizes the 30 carbon compound (triterpenoids), 24-methylene cholesterol (Figure 1B). Till date, complete information of whole withanolide biosynthesis pathway is not available. However, combination of several studies provide an overview of pathway illustrating several enzymatic steps (Mirjalili et al., 2009; Senthil et al., 2010; Chaurasiya et al., 2012; Gupta et al., 2013a,b, 2015; Dhar et al., 2015). Enzymatic steps of MVA and MEP pathways has been prescribed through the first transcriptome analysis of the plant (Senthil et al., 2010), which keeps improving with advancement in techniques (Gupta et al., 2013b, 2015; Senthil et al., 2015). These analyses reveal numbers of tissue specific unique sequences, differentially expressed genes related to biosynthesis of secondary metabolites.

### Genes involved in biosynthesis of withanolides

Genes involved in biosynthesis of withanolides are Δ14-sterol reductase (EC 1.3.1.70), 1-deoxy-D-xylulose-5-phosphate reducto-isomerase/reductase (DXR; EC 1.1.1.267), 1-deoxy-D-xylulose-5-phosphate synthase (DXS; EC 2.2.1.7), 2-C-methyl-D-erythritol 2,4-cyclodiphosphate synthase (MEcPP synthase, IspF, EC 4.6.1.12), 2-C-methyl-D-erythritol 4-phosphate cytidylyl transferase (EC 2.7.7.60), 3-hydroxy-3-methylglutaryl-coenzymeA reductase (HMGR; EC 1.1.1.34), 4-diphosphocytidyl-2-C-methyl-D-erythritol kinase (EC 2.7.1.148), 4-hydroxy-3-methylbut-2-enyldiphosphate reductase (EC 1.17.1.2), 4-hydroxy-3-methylbut-2-enyldiphosphate synthase (HMB-PPS, IspG, EC 1.17.7.1), acetyl-CoA acetyltransferase (ACT, EC 2.3.1.9), C-5-sterol desaturase (C5SD, EC 1.14.19.20), cycloartenol C-24 methyltransferase (EC 2.1.1.142), cycloartenol synthase (CAS; EC 5.4.99.8), cycloeucalenol cycloisomerase (EC 5.5.1.9), cytochrome-P450s reductase (CPR, EC 1.6.2.4), farnesyl diphosphate synthase (FPPS, EC 2.5.1.10), geranyl diphosphate synthase (GPPS, EC 2.5.1.1), geranyl-geranyl diphosphate synthase (GGPPS, EC 2.5.1.29), glycosyltransferases (GT, EC 2.4.-), hydroxymethyl glutaryl-CoA synthase (HMGS, EC 2.3.3.10), isopentenyl diphosphate isomerase (IPPI, EC 5.3.3.2), methyltransferase (MT, EC 2.1.1.), mevalonate diphosphosphate decarboxylase (EC 4.1.1.33), mevalonate kinase (MVAK, EC 2.7.1.36), obtusifoliol 14-demethylase (EC 1.14.13.70), phosphomevalonate kinase (EC 2.7.4.2), squalene synthase (SQS, EC 2.5.1.21), squalene monooxygenase/epoxidase (SQE, 1.14.14.17), sterol Δ7 reductase (DWF, EC 1.3.1.21), etc. (Senthil et al., 2010, 2015; Gupta et al., 2013b, 2015).

To understand the interactions of various molecular network in entirety, Dhar et al. (2015) and Singh et al. (2015b) summarized the available information of some *in vitro* studies with respect to regulation of pathway genes required for withanolide accumulation.

### Few important catalytic conversions of proposed pathways

Among a number of enzymes, SQS and SQE are considered as an important enzymes in the biosynthesis of triterpenoids. Considering this, Gupta et al. (2012) characterize isoforms of *SQS* gene, while, Razdan et al. (2013) perform characterization and promoter analysis of *SQE* gene from *W. somnifera*. To confirm the functional activity, both studies also involved the cloning, expression and purification of gens/enzymes in *E. coli*. Genes encoding DXS, DXR and HMGR enzymes expressed their importance by catalyzing the key regulatory step of the isoprenoid biosynthesis. These genes revealed tissue specific, chemotype specific and modulated expression while exposed to SA, MeJA, as well as MI (Akhtar et al., 2013; Gupta et al., 2013c).

Few members of sterol-GT (*SGT*) gene family of *W. somnifera*, have been recognized and characterized (Sharma et al., 2007; Madina et al., 2007a,b; Chaturvedi et al., 2012). *SGTs* are responsible for diversified glycosylation of sterols (including withanolides). The identified *SGT*s expressed different level of expression in different tissues as well as under different stress conditions, to proove their physiological importance (Sharma et al., 2007; Chaturvedi et al., 2011, 2012). Purified SGTs showed broad substrate specificity for sugar acceptor but not for the sugar donor (Madina et al., 2007a,b). Similar pattern of glycosylation was observed by Singh et al. (2013) during functional characterization of flavonoid-GT gene from *W. somnifera*.

### Variation in gene expression pattern according to tissue and stress conditions

Relation among few pathway genes, withanolides accumulation with morphogenic transition has been studied by Sabir et al. (2013). *In vitro* tissues belongs to different stages of organogenesis (rhizogenesis and shoot organogenesis) were used for the experiment. Accumulation of major withanolides and expression of HMGR, FPP synthase (FPPS), SQS, SQE, cycloaretenol synthase (CAS), GTs were analyzed at different morphogenic transition states.

Detailed study on four-CYP450 has been performed by Srivastava et al. (2015) to illustrate involvement of these enzyme in some specialized secondary metabolite (withanolides). The expression profiles of these CYPs showed chemotype-specific and tissue-specific variation, as well as variation in response to physiological and developmental factors. To expand the understanding of expression of genes in relation to withanolide biosynthetic pathway, Pal et al. (2016) perform experiments with different concentrations of fertilizers on fresh twigs of *W. somnifera*. Treated twigs related to highest accumulation of withaferin-A has been selected to analyse expression pattern of CYPs, allene oxide cyclases (AOCs) and few other pathway related genes.

## Tissue culture studies on *Withania somnifera*

### Seed germination in *W. somnifera*

Numerous, campylotropous, whitish, disk shaped seeds are found inside red or orange colored fruit (berry) of *W. somnifera*. Earlier reports mentioned high dormancy with poor seed viability (Khanna et al., 2013; Viji et al., 2013), also seeds of *W. somnifera* showed low and erratic germination with heterogeneous seedlings (Vashistha et al., 2010) having higher mortality rate of seedlings under field conditions (Khanna et al., 2013). The problems with seed germination of *W. somnifera* (*in vitro* and in field) guided the researchers toward finding of simple techniques with optimized conditions, in order to get faster and more germination rate. These conditions include nutrient medium, light conditions and condition of seeds, etc. These findings will help nursery workers and poor farmers interested in developing mass planting stock.

Soaking of seeds in water, diluted sodium hypochlorite, nitrate solutions (of potassium, ammonium, cobalt, sodium, calcium and zinc), has been suggested to soften the hard seed coat of *W. somnifera* (Kattimani and Reddy, 2001; Vashistha et al., 2010). Improved germination has been observed at 25 ± 2°C and 16-h-light/8-h-dark photoperiod with the light intensity of 3,000 lux (Kambizi et al., 2006; Khanna et al., 2013; Viji et al., 2013). In addition to these conditions, incision on seed coat and few pre-incubation conditions (dark or 15°C) increases the germination percentage (Pandey et al., 2013; Viji et al., 2013; Kumar et al., 2016).

### Regeneration and multiplication of *W. somnifera*

Seedlings, embryos, cotyledon, epicotyl, hypocotyl, petiole, leaves, nodes, internodes, stem, shoot tips and roots have been used in different experiments for callus induction, adventitious root induction, regeneration, differentiation, flower induction, and fruit setting (Sharada et al., 2008; Supe et al., 2011; Singh et al., 2017). Composition of gelling matrix was standardized for encapsulation of shoot tips of *W. somnifera* along with optimization of media composition (or soilrite) for conversion of encapsulated shoot tips into plantlets (Singh et al., 2006). Most studies with optimized *in vitro* tissue culture conditions of *W. somnifera* have been briefly summarized recently by Singh et al. (2017).

## Accumulation of withanolides in different types of *in vitro* culture

The ultimate goal of different studies on *W. somnifera* is to provide maximum and better plant material for therapeutic purpose. These involes standardization of phytochemical analysis of different types of tissues obtained from different region and accession of *W. somnifera*, for accumulation of therapeutic metabolites (Table 1). On the basis of difference in available withanolides, *W. somnifera* has been divided into various chemotypes (accessions). Differences in chemo-profile of some selected chemotypes have been documented in several studies (Dhar et al., 2006; Kumar et al., 2007; Scartezzini et al., 2007; Bhatia et al., 2013).

**Table 1:** Different conditions/situation in order to accumulate therapeutically important metabolites of *W. somnifera*.

		**Condition**	**Plant Part**	**Special treatment/condition/method/ identification**	**Metabolite extracted**	**References**
Standardization/ Identification of metabolic-analytical technique/ metabolite		*In situ*	Root; stem; leaf	HPLC for determination of withanolides	WS-1; WS-5	Ganzera et al., 2003
			Whole plant	cholinesterase inhibiting withanolides	2-new; 4- known withanolides	Choudhary et al., 2004
			Leaves	Sulfated and oxygenated withanolides	4-new; 6-known withanolides	Misra et al., 2005
			Dried roots/ leaves	HPLC and AFLP findings to relate different (15) accessions	WS-1; WS-2; WS-3; WS-7; WS-9; WSs; PG	Dhar et al., 2006
			Roots	Rare dimeric withanolide (ashwagandhanolide)	WS-1; WS-3; WS-7; WS-8; WS-14	Subbaraju et al., 2006
				Two new withanolides (TLC; NMR)	2-new and 7-known withanolides	Misra et al., 2008
			Leaves, roots	More reliable HPLC to determine broad range of withanolides	9- withanolides	Chaurasiya et al., 2008
			Various genotypes	HPTLC for determination of withanolides	WS-1; WS-3; WS-10	Srivastava et al., 2008
			Leaves, roots	NMR and HPLC and GC–MS for metabolic fingerprinting	48 to 62 primary/ secondary metabolite	Chatterjee et al., 2010
			Whole plant/ plant parts	Distribution in various organs	WS-3	Praveen et al., 2010
			Roots, fruits, leaves	Phenolic acids	5-phenolics; 3-flavonoids; few unknown	Alam et al., 2011
			Leaves, roots	HR-MAS-NMR to establish metabolic mapping (4 chemotypes)	41 metabolites	Bharti et al., 2011
Metabolic/ phytochemical profiling			Leaves, stems, roots	Metabolomic characterization (NMR) from different (6) regions	Primary and secondary metabolites	Namdeo et al., 2011
			Roots	Different species	21 bioactive compounds	Kumar et al., 2011
			Fruits	Developmental stages of fruit (NMR; COSYDQF; TOCSY; HSQC)	17 metabolites	Sidhu et al., 2011
				Fruits (LC-HRMS and LC-MS/MS)	62 metabolites	Bolleddula et al., 2012
				Chemotype (4) variations (GC–MS and NMR)	82 metabolites	Bhatia et al., 2013
			Leaves, roots	Clustering of accessions (25) based on phenotypic and chemotypic analysis	WS-1; WS-2; WS-3	Kumar et al., 2007
				Relation between transcript and metabolic profile in two morpho-chemovariant accessions	WS-1; WS-2; WS-3	Dhar et al., 2013
			Different plant parts	Growth dependent variation in few metabolites (2 cultivars)	WS-1; WS-2; WS-3; squalene	Dhar et al., 2016
Media/soil/elicitortreatment/variation		*In vitro*; *In situ*	Leaves	Nitsch and Nitsch-(NN) media + BAP + IBA	WS-1	Furmanowa et al., 2001
			Parts of seedlings	MS/ B5 basal media + (different combinations of plant hormones)	WS-1; WS-2; WS-3; WS-4; WS-6	Sharada et al., 2007
			Leaves, stem, roots	MS + BAP, IAA	WS-1; WS-3; WS-10	Dewir et al., 2010
			Leaves, roots, seedling	Sandy loam soil; MS	WS-1	Johny et al., 2015
		*In vitro*	Multiple shoots, teratoma	MS + BAP + Kinetin	WS-1; WS-3	Sangwan et al., 2007
			Multiple shoots	MS + BAP+/ IAA+/ IBA+/ NAA+/ 2,4-D	Glycowithanolides; withanolides	Ahuja et al., 2009
			Adventitious roots	MS + IBA + IAA	WS-3	Wasnik et al., 2009
				MS + 2,4-D/ IAA/ IBA/ NAA; B5 NN; N6	WS-3	Praveen and Murthy, 2010
			Adventitious roots from semi-friable callus of leaves	MS + 2,4-D + kinetin, MS + IBA + IAA	WS-1; WS-2; WS-3; WS-4; WS-10; WS-12; WS-13	Sivanandhan et al., 2012a
				MS + 2,4-D + kinetin, MS + IBA + NAA, Elicitors	WS-1; WS-3; WS-4; WS-12; WS-13	Sivanandhan et al., 2012b
			Plantlet	Hoagland + MeJA; SA	WS-1; WS-3	Rana et al., 2013
			Callus culture	MS + 2,4 D + kinetin	WS-1; WS-3	Chakraborty et al., 2013
			Adventitious root culture	MS + sucrose + IBA; different concentrations/ types of sugars; different pH	WS-3	Murthy and Praveen, 2013
			Cell suspension culture	MS + 40% *Gracilaria edulis* extract for 24 h	WS-1; WS-2; WS-3; WS-4	Sivanandhan et al., 2013b
			Multiple shoot cultures	MS + BAP + spermidine	WS-1; WS-2; WS-3; WS-4	Sivanandhan et al., 2013c
			Cell suspension culture	MS + kinetin + L-glutamine + sucrose + CaCl_2_/ NH_4_Cl/ chitosan/ cholesterol/ MA/ squalene	WS-1; WS-2; WS-3; WS-4; WS-11; WS-12; WS-13	Sivanandhan et al., 2014a
			Shoot suspension culture	MS + *Gracilaria edulis/ Sargassum wightii*	WS-1; WS-2; WS-3; WS-4	Sivanandhan et al., 2014b
			Flowers, fruits	MS + BAP + IAA, sucrose, L-glutamine, adenine sulfate, nitrates of ${\text{NH}}_{4}^{+}$, K^+^, Na^+^	WS-1; WS-2; WS-3; WS-4	Sivanandhan et al., 2015c
		*In situ*	Whole plant/ plant parts	Different vermicomposts	WS-1; WS-5	Raja and Veerakumari, 2013
			Leaves, roots	Organic composion of soil (bioaugmented organic + gypsum)	WS-1; WS-2; WS-3	Gupta et al., 2016
				SA; MeJA; MI (4 chemotypes)	WS-3	Gupta et al., 2011
		*In vitro*	*in vitro* culture	MeJA; GA3; YE	WS-1; WS-2; WS-3	Dhar et al., 2014
			Plantlet	MeJA; SA; GA3	WS-1; WS-2; WS-3	Rana et al., 2014
				MeJA; SA; 2,4-D; YE	WS-1; WS-3	Razdan et al., 2016
Strain-plasmid ± gene; tissue used for infection of	*A. rhizogene*	*In vitro*	Hairy roots	LBA 9402 -pRi 1855; stem, leaves	WS-5	Ray et al., 1996
				MTCC 2364, MTCC532; stem, hypocotyle, leaves	Not mentioned	Pawar and Maheshwari, 2004
				LBA 9402; A4-pRiA4; leaves	WS-1; WS-5	Bandyopadhyay et al., 2007
				R1601- pRiA4b; different parts of seedling	WS-3	Murthy et al., 2008
				LBA9402/ A4 ± synthetic crypt gene; leaves	WS-1	Chaudhuri et al., 2009
				15834; leaves	WS-1; WS-3	Doma et al., 2012
				ATCC 15834, R1000, K599; leaves, petiole, internodes	WS-1	Saravanakumar et al., 2012
				R1601; cotyledonary leaves	WS-3	Praveen and Murthy, 2013
				R1000; leaves	WS-1; WS-2; WS-3	Sivanandhan et al., 2013a
				A4 ± SGT; leaves	WS-3	Pandey et al., 2015
				LBA9402 ±β-cryptogein gene; leaves	WS-1; WS-3	Sil et al., 2015
				leaves	WS-1; WS-2; WS-3; WS-4	Sivanandhan et al., 2015b
				R1000, MTCC 2364, MTCC 532*;* leaves	WS-1; WS-3	Thilip et al., 2015
	*A. tumefaciens*	*In vitro*	Teratoma	Nopaline:C58; octopine:Ach5, disarmed:LBA 4404; leaves	WS-1; WS-5	Ray and Jha, 1999
			Plantlet	GV3102 - pIG121Hm ±CAS gene/ pGSA1131 ±RNAi; leaves	Total withanolide	Mishra et al., 2016
		*In situ*	Leaves	GV3102- pBI121 ±*WsSQS*; leaves	WS-3	Grover et al., 2013
				*Agroinfiltration* (GV2260- pCAMBIA ±WsSQS) ± Microprojectile; leaves	WS-1; WS-2; WS-3; WS-4	Patel et al., 2014, 2015
				LBA4404/GV3102 - pFGC1008/pBI121/TRV2/ ±SGT gene/s; leaves	WS-1; WS-2; WS-3; WS-13	Saema et al., 2015, 2016; Singh et al., 2016
				LBA4404-pCAMBIA; leaves	WS-1; WS-2; WS-3; WS-4	Sivanandhan et al., 2015a

*HPLC, high performance liquid chromatography; HPTLC, High performance thin layer chromatography; TLC, Thin layer chromatography; LC-MS, Liquid chromatography-mass spectrometry; NMR, Nuclear magnetic resonance; GC-MS, Gas chromatography mass spectrometry; FAB, Fast atom bombardment; HRMS, high resolution mass spectroscopy; COSYDQF, Two-dimensional (2D) phase-sensitive double quantum filtered correlation spectroscopy; TOCSY, Total correlation spectroscopy; HSQC, ^1^H–^13^C hetero nuclear single quantum correlation; HR-MAS-NMR, High Resolution Magic Angle Spinning-NMR; PCA, principal component analysis; HCA, hierarchical clustering analysis; MA, mevalonic acid; WS-1, withaferin A; WS-2, withanone; WS-3, Withanolide-A; WS-4, Withanolide-B; WS-5, Withanolide-D; Ws-6, withanolide-E; WS-7, 27-hydroxywithanone; WS-8, 20-deoxywithanolide A; WS-9, withastramonolide; WS-10, 12-deoxywithastramonolide; WS-11, 12 deoxy withanstramonolide; WS-12, withanoside-IV; WS-13, withanoside-V; WS-14, ashwagandhanolide; WSs, withanosides; PG, physagulin*.

Variation persist in accumulation of withanolides due to plant parts, developmental stages (Praveen and Murthy, 2010; Dhar et al., 2013), plant part obtained from different types of cultures (Sharada et al., 2007; Singh et al., 2017) of *W. somnifera*. These studies establish relationship between morphology/condition of plant tissue and withanolide contents. Sivanandhan et al., 2012a,b, 2013b,c, 2014a,b, 2015a; Singh et al., 2017) used *in vitro* grown plants in different studies to develop adventitious roots, multiple shoots, shoot suspension culture, cell suspension culture, flowers, and fruits using different growth conditions. These developed tissues were harvested to extract different combinations of withanolides.

Based on different studies, Singh et al. (2017) summarized effects of *in vitro* conditions on accumulation of withanolides. These studies involving organ and callus culture, cell suspension culture and *Agrobacterium tumefaciens* as well as *A. rhizogene* mediated transformation. Different conditions of these techniques resulted in modulated accumulation of different withanolides, some of which related to modulated gene expression pattern.

### Hairy root culture of *W. somnifera* and withanolide accumulation

Hairy root cultures are a promising approach of bioprocess engineering for large scale production of valuable plant secondary metabolites. There are several reports available in order to modulate quantity of withanolides in hairy roots culture using *A. rhizogenes* mediated transformation (Pawar and Maheshwari, 2004; Bandyopadhyay et al., 2007; Murthy et al., 2008; Saravanakumar et al., 2012; Sivanandhan et al., 2013a, 2015b). It has been reported that different factors like carbohydrates (Doma et al., 2012), inorganic supplements (Praveen and Murthy, 2013), seaweed extracts (*Gracilaria edulis* and *Sargassum wightii*; Sivanandhan et al., 2015b), hormones, elicitation (like, chitosan, JA, SA; Chaudhuri et al., 2009; Doma et al., 2012; Sivanandhan et al., 2013a), etc. modulate biogeneration of withanolides in hairy root cultures.

Difference in hairy root emergence was observed illustrating resistance or susceptibility of *W. somnifera* toward different strains of *A. rhizogenes* (Pawar and Maheshwari, 2004; Bandyopadhyay et al., 2007; Saravanakumar et al., 2012) as well as transformation efficiency of different explants used for the experiment (Murthy et al., 2008; Saravanakumar et al., 2012). Leaves proved to be more appropriate for infection by different strains of *A. rhizogene*, since used as explant in various studies (Ray et al., 1996; Bandyopadhyay et al., 2007; Chaudhuri et al., 2009; Doma et al., 2012; Saravanakumar et al., 2012; Praveen and Murthy, 2013; Sil et al., 2015; Thilip et al., 2015). Recenlty, Pandey et al. (2015) induced hairy root from leaf explants of *W. somnifera* expressing *sterol glucosyltransferase* gene (clone-4) using *A. rhizogenes*. The transgenic hairy roots were observed to accumulate higher amount of withanolide-A when subjected to elicitation (salicylic acid and methyl jasmonate).

### *A. tumefaciens* mediated transformation and its application to modulated withanolide biosynthesis

Numerous studies have helped in developing efficient methods for regeneration of *W. somnifera*, while only few reports are available for genetic transformation for this medicinal plant (Singh et al., 2017). Altered expressions of genes related to biosynthetic pathway, ultimately modulate quantity of plant secondary metabolites, which are of therapeutic importance. Ray and Jha (1999) infected leaves of *in vitro* grown plants (two genotypes) with wild type nopaline and octopine strains of *A. tumefaciens*. Different types of galls obtained due to different levels of virulence on the two genotypes. Two principle withanolides, withanolide D and withaferin A extracted from shooty teratoma cultures in higher amount, while, withanolide D alone was detected in rooty teratomas.

Pandey et al. (2010) performed successful *A. tumefaciens* mediated transformation with 1.67 efficiency using non-virulent strain. Leaves excised from 1-5-nodes of both *in situ* and *in vitro* grown 30 to 90-day-old seedlings of different accessions of *W. somnifera* were used for the study. LBA4404 containing the binary vector pIG121Hm showed more gus expression in second and third leaves of 75 day old seedlings. Leaf explants ultrasonicated at 47 KHz ± 6% for 10 s showed higher gus expression as compared to directly infected explants. The protocol was used to analyse *in vivo* enzymatic action of one SGT (*WsSGTL1*) of *W. somnifera* by Saema et al. (2015, 2016). RNAi silencing (Saema et al., 2015) as well as overexpression (Saema et al., 2016) of *WsSGTL1* gene has been achieved in transgenic *W. somnifera*. As expected, reduction in the level of glycosylated products observed in transgenic with silenced *WsSGTL1* transcript. However, transgenics with overexpressing *WsSGTL1* showed early and enhanced growth, increased production of glycosterols, and glycowithanolides. These transgenics displayed biotic (*Spodoptera litura*) and abiotic (cold) stress tolerance as well as recovery after cold stress along with improved photosynthetic performance.

Patel et al. (2014) established *A. tumefaciens* mediated transformation, microprojectile bombardment and microprojectile bombardment assisted agroinfection. Apical and nodal explants obtained from multiplied culture after *in vitro* seed germination were used as explants. Modified vector pCAMBIA1301 used to confirm transgene expression. Pre-cultured explants were bombarded and immediately infected with *A. tumefaciens* for microprojectile bombardment assisted agroinfection. The transformation efficiencies achieved were 3.86, 3.62, and 8.71%, through *A. tumefaciens* mediated, microprojectile bombardment and with the combination of both, respectively.

The protocol (Patel et al., 2014) used to overexpress of *WsSQS* in *W. somnifera* (Patel et al., 2015). Grover et al. (2013) also transformed leaves and shoots of 4-6-weeks old seedlings with *A. tumefaciens* (GV3101 harboring pBI121H) containing *SQS* from *W. somnifera*. Transgenics were confirmed with enhance expression of *WsSQS* transcript and its enzymatic activity. Higher amount of different withanolides observed in transgenics to proove the involvement of SQS with enhanced withanolide biosynthesis.

Nodal explants of 3-month old filed grown plants were used to develop transformation protocol of *W. somnifera* by Sivanandhan et al. (2015a) with 10% efficiency. These explant were found as an ideal tissue for the production of higher number of multiple shoots, hence adopted for the production transgenics. Explants were precultured (6-days) to obtain maximum transformation efficiency using *Agrobacterium* suspension (strain LBA4404 harboring pCAMIBA2301) at 0.2 OD_600_. The transformation frequency increased significantly with wounded nodal explants subjected to a sonication (10 s, longer treatment affected the viability of regenerating cells). Maximum transformation efficiency of 10.6% was observed by Mishra et al. (2015) using nodal explants infected with *A. tumefaciens* strain GV3101 harboring pIG121Hm. Explants were pre-cultured on MS supplemented with TDZ for 2 days and infected with *Agrobacterium* (0.2 OD_600_) for 20 min and co-cultivated for 48 h at 22°C.

Virus induced gene silencing methods was adopted by several researchers to achive fast and efficient characterization of genes related to withanolide biosynthesis. Using this technology, successful silencing of SQS (Singh et al., 2015a), *WsDWF-5* (Gupta et al., 2015) and three*-WsSGTLs* genes (Singh et al., 2016) were achived in *W. somnifera*. *Ws-SQS* silenced plants revealed positive and negative affects on expression of upstream asnd downstream pathway genes, which ultimately reduces the accumulation of phytosterols. Silencincing of *WsDWF-5* was observed with reduced accumulation of withanolide while, 3-*WsSGTLs* gene silencing found associated with enhanced level of different withanolides and reduced level of glycowithanolides. Increased expression of other upstream genes of withanolide biosynthesis pathway also relates with the supressed activity of *WsSGTLs*, which leads to reduced tolerance toward biotic stress.

## Conclusion

*W. somnifera* is of great importance in lots of medical conditions due to abundance of diversified therapeutic secondary metabolites (withanolides). Significance of the plant leads researchers to identify the best suitable way to enhace plant productivity according to increasing demands. In order to complete the requirement, complete information related to metabolites, their biosynthesis (pathway genes/enzymes) and effect of different factors (composition of soil/media, elicitors etc.) is essential. Under the influence of significance of biosynthetic pathway, related genes/enzymes and external factors, this review describes all analyzed combinations of molecular and/or *in vitro* techniques that modifies the accumulation of desired metabolites. Several environmental factors like, soil/media composition, different types of elicitors/stresses etc. affect the withanolide biosynthesis by regulation of gene expression pattern. A lot of investigations included in this review that analyse withanolide accumulation through different types of *in vitro* culture techniques, like, micropropagation, organogenesis, hairy root production etc. Combination of optimized *in vitro* techniques and information of pathway gene/enzyme are of great interest these days. Such combination of genetic transformation and optimized *in vitro* conditions provides much better productivity in terms of metabolite accumulation. The present review describes that there are a lot more combinations available and need to utilize in order to achieve best productivity, to make it easily accessible for the progress of medical industry.

## Author contributions

VP and WA collected literature and wrote the manuscript, PM and NA critically evaluated the manuscript. All authors approved the manuscript.

### Conflict of interest statement

The authors declare that the research was conducted in the absence of any commercial or financial relationships that could be construed as a potential conflict of interest.

## Abbreviations

SA: salicylic acid
MeJA: methyl jasmonate
MI: mechanical injury
ABA: abscisic acid
JA: jasmonic acid
*Ws*: *Withania somnifera*
GA3: gibberellic acid
YE: yeast extract.
